# Non-technical skills in acute care: an umbrella review of assessment, training, and implications for emergency department practice

**DOI:** 10.3389/fmed.2026.1791714

**Published:** 2026-04-28

**Authors:** Yukun Zhang, LiYoong Tang, MeiChan Chong, Xihui Sun, Manyu Jiang

**Affiliations:** 1 Department of Nursing, The Second Affiliated Hospital of Zhejiang University School of Medicine, Hangzhou, Zhejiang, China; 2 Department of Nursing Science, Faculty of Medicine, Universiti Malaya, Kuala Lumpur, Malaysia

**Keywords:** assessment instruments, emergency department practice, healthcare professionals, non-technical skills, simulation-based training, umbrella review

## Abstract

**Background:**

Non-technical skills (NTS), including communication, teamwork, leadership, situational awareness, and decision-making, are widely recognized as essential for safe and effective performance in high-risk healthcare environments. Although extensive research has examined NTS training and assessment across healthcare settings, the evidence base is fragmented, and its applicability to emergency department (ED) practice remains unclear. This umbrella review synthesizes review-level evidence on NTS assessment tools and training approaches in healthcare and examines their implications for emergency department practice.

**Methods:**

A systematic search was conducted across PubMed (MEDLINE), Scopus, Cochrane Library, Web of Science, and EBSCOhost (CINAHL, APA PsycInfo, and Academic Search Complete) from inception to February 2025. Eligible studies included systematic, scoping, and narrative reviews examining the assessment, training, or implementation of NTS among healthcare professionals. The review protocol was registered with PROSPERO (CRD420251008161). Data were extracted and synthesized using a structured narrative synthesis, with explicit consideration of relevance and transferability to emergency department contexts.

**Results:**

A total of 2,229 records were identified, and 17 reviews published between 2012 and 2024 were included, with 88% originating from high-income countries. Key themes encompassed NTS conceptual frameworks, methodological approaches, assessment instruments, and outcome measures. Widely reported frameworks and tools included Crisis Resource Management, Trauma Non-Technical Skills (T-NOTECHS), and the Team Emergency Assessment Measure (TEAM). Evidence most consistently indicated improvements in team behaviors and process performance, predominantly in simulation-based or observational settings. No meta-analyses were identified.

**Conclusions:**

This umbrella review synthesizes review-level evidence on NTS assessment and training across healthcare and highlights considerations for application in emergency department practice. The evidence most consistently supports improvements in team behaviors and process performance, largely in simulation-based or observational settings, while direct evidence for effects on patient outcomes in routine ED care remains limited. Future research should prioritize multicentre, cross-cultural, and longitudinal designs with harmonized outcomes to better evaluate implementation, effectiveness, and contextual transferability to emergency department settings.

## 1 Introduction

The emergency department (ED) is a fast-paced and high-stress clinical environment in which rapid and accurate clinical decisions are often required (1). Characterized by unpredictable patient volumes, undifferentiated presentations, and the need for multidisciplinary coordination, emergency care relies not only on clinical knowledge and technical expertise but also on non-technical skills (NTS). These include communication, teamwork, situational awareness, decision-making, leadership, and stress management, which support information sharing, coordination, and adaptive responses during time-critical care (2, 3).

Across acute healthcare settings, the importance of NTS is increasingly recognized (4). Effective communication, sound decision-making under uncertainty, and collaboration within multidisciplinary teams have been shown to influence care quality and efficiency across a range of clinical contexts, including emergency care (5). Deficits in NTS, particularly communication breakdowns and failures in coordination, have been repeatedly identified as contributing factors in adverse events and safety incidents in acute care (6, 7). In response, structured approaches to NTS training, such as crisis resource management, have been developed and widely applied in healthcare. These approaches draw on principles established in high-reliability industries, including aviation and nuclear power (8, 9). However, the extent to which such approaches have been consistently integrated, assessed, and sustained within routine emergency department practice remains unclear (10).

The literature addressing NTS in relation to emergency medicine is heterogeneous and often indirect. Many studies focus on individual skill domains, specific clinical activities such as trauma or resuscitation, or healthcare professionals more broadly, rather than on emergency departments as a distinct organizational and clinical setting. For example, Singh et al. (11) examined situational awareness in relation to diagnostic performance, while Kern et al. (12) explored the influence of leadership on team performance during resuscitation. In addition, inconsistent terminology, including the use of terms such as non-technical skills, soft skills, and human factors, together with variability in assessment approaches, complicates synthesis and limits the translation of findings into routine emergency department practice (13).

An umbrella review, defined as a review of reviews, provides a structured approach to consolidating and appraising evidence synthesized across multiple reviews, identifying patterns and gaps across heterogeneous evidence bases, and supporting translation to practice (14, 15).

### 1.1 Aim

This umbrella review aimed to synthesize review-level evidence on the assessment, training, and implementation of NTS across healthcare settings, with a specific focus on examining the implications and potential relevance of this evidence for emergency department practice.

## 2 Materials and methods

### 2.1 Study design and methodological approach

The conduct of this umbrella review was guided by the methodological framework outlined in the Joanna Briggs Institute Manual for Evidence Synthesis (16). The review protocol was prospectively registered in PROSPERO (CRD420251008161). Reporting followed the PRISMA 2020 statement, adapted for umbrella reviews (17). Given that the aim was to consolidate review-level evidence on NTS across acute and high-risk healthcare contexts, we included systematic reviews as well as other review types, including scoping reviews, mapping reviews, and narrative or critical reviews when they provided relevant synthesis on NTS conceptualization, assessment, training, or implementation. Accordingly, methodological quality or reporting completeness was appraised using tools appropriate to each review type (AMSTAR 2 for systematic reviews; PRISMA-ScR as a reporting checklist for scoping reviews; and SANRA for narrative reviews).

To address redundancy across reviews, we constructed a citation matrix of primary studies and quantified overlap using the corrected covered area (CCA) method. Overlap estimates were interpreted descriptively and used to inform cautious interpretation of recurring findings driven by shared primary studies.

### 2.2 Ethics approval

All data used in this review were sourced from publicly accessible publications. As such, this study did not involve human subjects and was not classified as human research, thus ethics committee approval was not required.

### 2.3 Search strategy

An extensive literature search was conducted in five major electronic databases, including PubMed (MEDLINE), Scopus, Cochrane Library, Web of Science, and EBSCOhost (CINAHL, APA PsycINFO, and Academic Search Complete), from inception to February 2025 (see Supplementary Additional File 1). To ensure currency, we conducted an update search on 15 January 2026 using the same databases and search strategy as the original search (from inception to February 2025). No additional eligible reviews were identified between February 2025 and the update-search date. The search strategy was designed to identify review-level evidence on the assessment, training, and implications of NTS among healthcare professionals.

To reflect the heterogeneous terminology used in this field, the search extended beyond the term NTS to include related constructs and commonly used descriptors such as human factors, teamwork, communication, leadership, situational awareness, decision-making, and crisis resource management. Database specific subject headings and free-text terms were combined as appropriate. Rather than restricting retrieval to emergency department settings, healthcare context terms were used broadly to capture evidence from acute and high-risk clinical environments. To support assessment of relevance to emergency department practice, emergency care related terms such as emergency department, emergency medicine, acute care, and resuscitation were incorporated where applicable. The search strategy was iteratively refined through pilot testing to improve sensitivity and relevance. To further minimize the risk of missing eligible reviews, we searched additional sources (including ProQuest Dissertations and Theses Global) and conducted reference list screening and forward citation tracking of included reviews using Google Scholar and Epistemonikos. Where available, gray literature sources were also checked. Where necessary, authors of conference abstracts were contacted to obtain full texts (18).

### 2.4 Eligibility criteria

Eligibility criteria for inclusion are summarized in Table 1. We included systematic reviews, scoping reviews, mapping reviews, or narrative reviews that examined the assessment, training, or implementation of NTS among healthcare professionals. Primary research studies, editorials, opinion pieces, conference abstracts without full texts, and reviews not addressing NTS were excluded.

**Table 1:** Eligibility criteria.

Eligibility criteria	Description
Study design	Systematic reviews, scoping reviews, mapping reviews, or narrative reviews.
Population	Healthcare professionals involved in acute or high-risk clinical care, including nurses, physicians, paramedics, and other members of multidisciplinary healthcare teams.
Setting	Healthcare settings in which team-based, time-critical care is delivered, including emergency departments as well as other acute care environments such as resuscitation, trauma care, and simulation-based training contexts.
Intervention	Reviews examining the assessment, training, or implementation of non-technical skills, including their association with team behaviors, process performance, or safety-related outcomes.
Outcomes	Outcomes related to non-technical skill domains relevant to emergency department practice, such as communication, teamwork, decision-making, situational awareness, leadership, and stress management.
Languages	Published in peer-reviewed journals in English.

### 2.5 Study selection

Study selection followed PRISMA guidance. After automatic and manual removal of duplicates in EndNote, two reviewers independently screened titles and abstracts, followed by full-text assessment against the predefined eligibility criteria. Consistent with the review objective, studies were not restricted to emergency department settings at the screening stage, provided that the review focus was relevant to NTS in acute or high-risk healthcare contexts. Disagreements were resolved through discussion, with a third reviewer consulted when consensus could not be achieved.

### 2.6 Quality assessment

We assessed the methodological quality or reporting completeness of included reviews using tools appropriate to review type. Systematic reviews were appraised using AMSTAR 2 (19), with overall confidence classified as high, moderate, low, or critically low based on critical-item performance. Scoping reviews were assessed for reporting completeness using the PRISMA-ScR checklist (20). Narrative and critical reviews were evaluated using the SANRA instrument (21). Assessments were performed independently by two reviewers, with disagreements resolved through discussion and consultation with a third reviewer when required. These assessments informed the interpretation of findings, with more cautious inference drawn from reviews with lower methodological confidence or incomplete reporting.

### 2.7 Data extraction

Following eligibility confirmation, a pilot-tested and standardized data extraction form was used to record key characteristics of each included review, including author, publication year, review aim, review design, time span covered, main findings, and reported outcome measures. Information relevant to the context of care and potential applicability to emergency department practice was also extracted where reported. Data extraction was conducted independently by two reviewers, with discrepancies resolved through discussion and adjudication by a third reviewer when consensus could not be reached.

### 2.8 Data synthesis

Guided by the Joanna Briggs Institute methodology for umbrella reviews (16), a structured narrative synthesis was undertaken. Evidence from the included reviews was organized thematically, with attention to NTS domains, assessment approaches, and training strategies, as well as their reported relevance or potential applicability to emergency department practice. Outcomes were presented in summary tables and further elaborated in the main text.

## 3 Results

**Figure 1:**
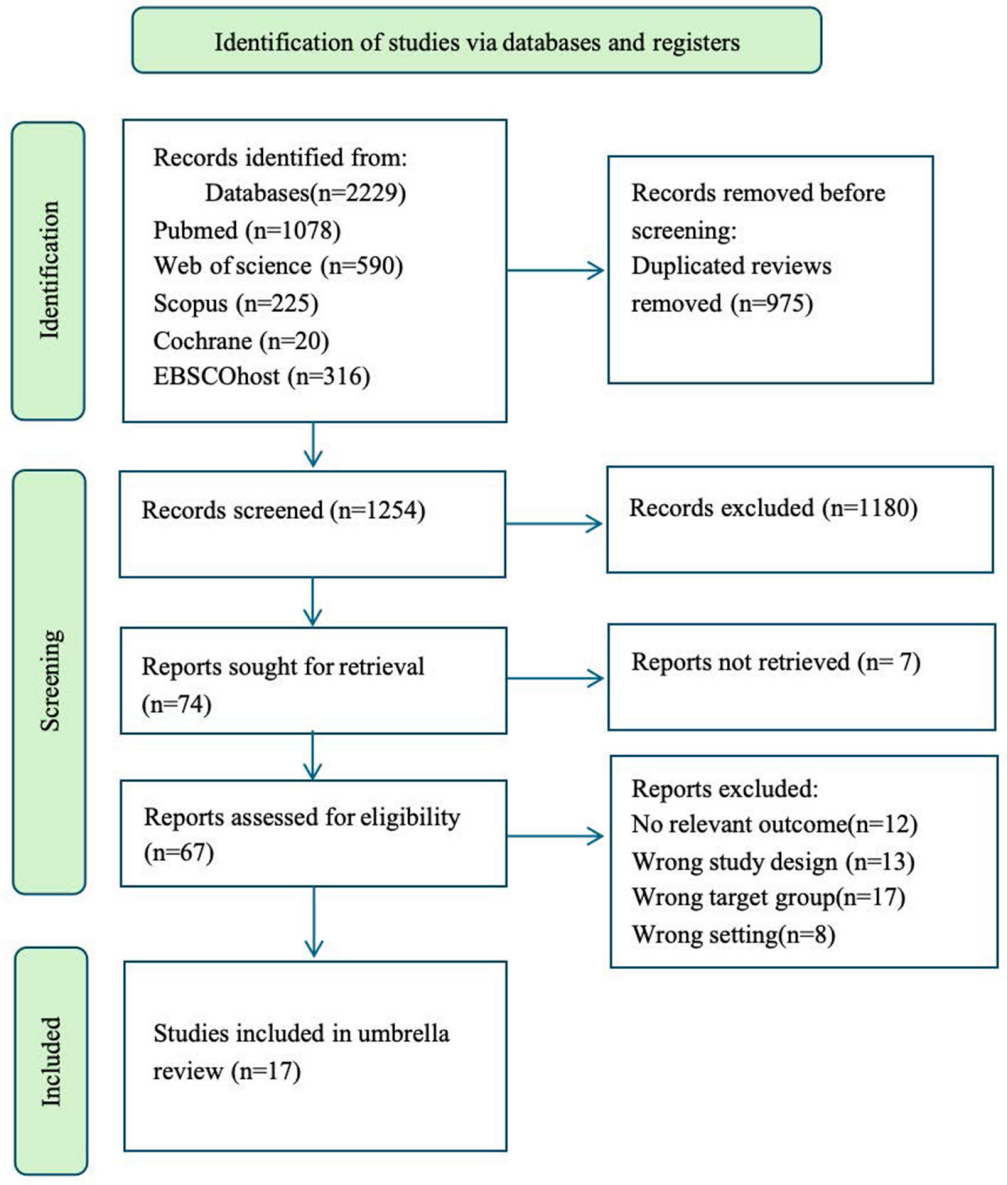
PRISMA flow diagram.

The database search yielded 2,229 records. After removal of 975 duplicates, 1,254 unique records remained. Title and abstract screening excluded 1,180 records that did not meet the eligibility criteria, leaving 74 articles for full-text assessment. Of these, 50 were excluded because they did not address NTS assessment, training, or implementation, and 7 could not be retrieved despite repeated attempts, including institutional access checks, interlibrary loan requests, and direct author contact. These records were primarily conference abstracts or reports without accessible full texts through the searched databases. Consequently, 17 reviews were included in the final synthesis. None of the included reviews reported a meta-analysis. The study selection process is illustrated in Figure 1.

### 3.1 Characteristics of the included studies

The 17 included reviews were published between 2012 and 2024, with the majority published from 2020 onwards (14/17, 82%). Collectively, these reviews examined core NTS domains relevant to team-based care in acute and high-risk healthcare settings, including communication, teamwork, leadership, clinical decision-making, situational awareness, and stress management, across a range of clinical contexts.

To assess potential redundancy, we conducted a formal overlap analysis of primary studies included across reviews. The analysis indicated a moderate degree of overlap [Corrected Covered Area (CCA) = 8.4%], primarily attributable to repeated inclusion of validation and application studies of widely used NTS assessment instruments, particularly the Team Emergency Assessment Measure (TEAM) and the Trauma Non-Technical Skills scale (T-NOTECHS). Beyond these instrument-focused clusters, overlap was limited, suggesting that most reviews contributed complementary rather than redundant perspectives to the overall evidence base.

Fifteen reviews (88%) originated from high-income countries, most commonly the United Kingdom (*n* = 4), Australia (*n* = 4), and Canada (*n* = 2). Two reviews (12%) incorporated evidence from low- and middle-income countries (LMICs). Across the 17 reviews, the number of primary studies synthesized ranged from 5 to 215, comprising 828 unique primary studies in total. The included reviews addressed emergency and resuscitation teams (22–28), physicians (29, 30), nurses (31, 32), paramedics (33), and multidisciplinary professionals in acute care settings (34). Six reviews (23, 24, 31, 35–37) reported that most of their included evidence was derived from simulation-based settings, including high-fidelity simulation. Reported outcomes mainly focused on team behaviors and process-related performance indicators. Table 2 summarizes key assessment tools, training approaches, and quantitative evidence reported in the included reviews and their relevance to emergency department practice. Table 3 presents the characteristics of the 17 included reviews, and Table 4 provides an evidence map of review focus, study settings, and outcome types across the evidence base.

**Table 2:** Key non-technical skills assessment tools, training evidence, and relevance to emergency department practice.

Evidence category	Tool/training approach	Key evidence synthesized in included reviews	Quantitative evidence reported in reviews	Relevance to emergency department practice
Team performance assessment	Team Emergency Assessment Measure (TEAM)	Widely applied across emergency teams, particularly in simulation-based studies, to assess leadership, teamwork, and task management (23, 31).	Primary studies summarized in Cooper et al. (23) reported improvements in TEAM global scores after structured training (approximately 6.0 to 9.0), with effect sizes ranging from Cohen's *d* = 0.61–1.21	TEAM may support structured evaluation of team performance during simulation-based resuscitation training and team debriefing in emergency departments
Team performance assessment	Trauma Non-Technical Skills (T-NOTECHS)	Commonly used for assessment of trauma and resuscitation teams, particularly in observational or video-based team performance review (22, 28).	Evidence from trauma team studies reviewed in Alexander et al. (22) supports the use of T-NOTECHS for structured video-based evaluation of resuscitation teams.	T-NOTECHS may be useful for retrospective team performance review in trauma and resuscitation cases, particularly when video recording is available.
Behavioral marker systems	Anesthetists' Non-Technical Skills (ANTS)	Behavioral marker system developed for anesthesia training and assessment, with structured domains including task management, teamwork, situational awareness, and decision-making (34).	Systematic review evidence primarily reports psychometric and measurement properties rather than intervention effect estimates (34).	Behavioral marker frameworks such as ANTS may inform the development of structured NTS training curricula for emergency teams but require contextual adaptation.
Simulation-based NTS training	Crisis resource management (CRM) and high-fidelity simulation	Simulation-based team training with structured debriefing is frequently used to develop NTS in emergency and resuscitation teams (24, 36).	Dewolf et al. (24) reported reductions in time to completion of simulated cardiac arrest scenarios and improved team behaviors; behavioral improvements were maintained for approximately 3–6 months in some studies.	Simulation combined with structured debriefing may support development of communication, leadership, and coordination skills in emergency resuscitation teams.
Alternative training formats	Escape room–based CRM training	Escape room–based educational strategies have been explored as experiential training approaches for teamwork and crisis resource management (35).	Evidence remains limited; Jaspers et al. (35) reported insufficient data to determine how specific design features influence learning outcomes.	Alternative experiential training approaches may support engagement in NTS training but require further evaluation of effectiveness.
Role-specific NTS competencies	Paramedic NTS frameworks	Scoping review evidence identified teamwork, communication, situational awareness, and decision-making as key competencies for paramedic practice (33)	Quantitative intervention evidence not reported; findings derived from competency mapping	Findings may inform role-specific training requirements for prehospital and emergency team members
Stress and team dynamics	Shared mental models and communication strategies	Stress negatively affected team performance processes, including decision-making, communication, and task execution (27).	Evidence synthesized narratively; quantitative intervention data not reported.	Structured communication strategies and shared mental models may help maintain team coordination during high-stress emergency scenarios.

**Table 3:** Characteristics of included reviews.

References	Aim	Type of review	Period covered	Key contribution
Carne et al. (29)	To outline core principles of crisis resource management (CRM) in healthcare and appraise their applicability to emergency medicine.	Narrative review	From inception until November 2010	Described core crisis resource management principles and discussed their relevance for emergency medicine training and practice.
Jepsen et al. (26)	To review the development and use of behavioral rating tools for assessing NTS at individual and team levels across clinical specialties and settings.	Critical review	Not mentioned	Identified behavioral rating instruments used to assess non-technical skills at individual and team levels in healthcare.
Higham et al. (37)	To synthesize observer-based instruments that assess NTS for healthcare professionals and students in simulation and clinical practice; to summarize development processes; and to appraise validity and usability evidence.	Systematic review	From January 1990 to March 2018	Synthesized observer-based instruments used to assess non-technical skills and examined their development processes, validity evidence, and usability.
Dewolf et al. (24)	To determine whether instruction targeting NTS improves performance during advanced life support (ALS) simulations.	Systematic review	From inception until August 1, 2018	Evaluated whether NTS-focused training improves performance during advanced life support simulations.
Bennett et al. (33)	To identify NTS that have been empirically examined in paramedicine and to compile the first evidence-informed list of desirable NTS for paramedics.	Scoping review	From inception until March 2020	Mapped empirically studied non-technical skills and identified competencies relevant to paramedic practice.
Evans et al. (25)	To clarify team dynamics and establish a shared terminology for NTS in *ad hoc* prehospital, emergency department, and trauma resuscitation teams, with the goal of guiding future research and education.	Scoping review using PRISMA-ScR and Arksey & O'Malley's framework	From inception until October 12, 2021	Reviewed team dynamics in *ad hoc* emergency and trauma teams and proposed a taxonomy describing NTS-related constructs in resuscitation settings.
Hu et al. (38)	To synthesize the characteristics and shared elements of non-technical competency frameworks for health professionals working in emergency and disaster settings.	Systematic review	From inception until March 2018	Synthesized competency frameworks for emergency and disaster health professionals and examined variation in framework domains and terminology.
Tan et al. (30)	To identify the NTS required by junior doctors to manage acute medical emergencies.	Scoping review	From 2000 to March 2020	Identified the non-technical skills required by junior doctors managing acute medical emergencies.
Gawronski et al. (31)	To summarize the domains, item structures, and psychometric properties of instruments developed to evaluate healthcare providers' NTS during high-fidelity simulation (HFS).	Systematic review	From inception until September 2022	Reviewed instruments used to evaluate healthcare providers' non-technical skills in high-fidelity simulation settings.
Stevenson et al. (28)	To critically review the development process of the T-NOTECHS instrument and summarize existing evidence regarding its measurement properties, including sensibility, reliability, and validity.	Scoping review	From inception until July 8, 2021	Examined the development process and measurement properties of the T-NOTECHS instrument used in trauma team assessment.
Alexander et al. (22)	To review instruments used to assess NTS in emergency department resuscitation teams via video analysis and to evaluate the supporting evidence for their validity and practical usability.	Systematic review	From January 1995 to September 2023	Evaluated instruments used to assess non-technical skills in emergency department resuscitation teams using video analysis.
Cooper et al. (23)	To explore how the Team Emergency Assessment Measure (TEAM) has been applied to enhance emergency team NTS over the past decade.	Mapping review	From 2012 to 2022	Reviewed the application of the TEAM instrument for assessing non-technical performance in emergency team settings.
Zhang (36)	To summarize insights gained over two decades from the use of medical simulation to train NTS in emergency medicine.	Narrative review	From 2001 to 2020	Summarized simulation-based approaches used to develop non-technical skills in emergency medicine.
Jaspers et al. (35)	To identify shared design characteristics of escape rooms used for CRM and teamwork training in acute care, examine their relationship with learning outcomes, and propose evidence-based design requirements.	Scoping review	From 2000 to June 2023	Reviewed the design characteristics and educational use of escape room-based teamwork and crisis resource management training.
Kang et al. (34)	To synthesize evidence on the development and application of the Anesthetists' Non-Technical Skills (ANTS) behavioral marker system as a tool for training and assessing NTS to enhance patient safety.	Systematic review	From 2002 to May 2022	Synthesized evidence on the development and application of the ANTS framework.
Rheinberger et al. (32)	To identify, consolidate, and summarize evidence on the training needs for clinical and NTS required of emergency resuscitation nurses.	Scoping review	From inception until June 2024	Summarized clinical and non-technical competencies required for emergency department resuscitation nurses.
Sorensen et al. (27)	To synthesize evidence on how stress influences team performance in interprofessional healthcare teams.	Narrative review	From 1 January 1990 to 16 August 2021	Reviewed how stress influences team processes and performance in interprofessional healthcare teams.

**Table 4:** Evidence map of included reviews.

References	Type of review	Primary focus	Setting explicitly described	Main outcomes synthesized	Quantitative outcome data reported in the review
Carne et al. (29)	Narrative review	Framework/Conceptual (CRM)	Emergency medicine (practice/training context), mixed/unspecified	Conceptual principles; implementation/credentialing advocacy	Limited/No
Jepsen et al. (26)	Critical review	Assessment tools/measurement	Across specialties; mixed/unspecified	Inventory of behavioral rating instruments; need for validation/rater training	Yes (psychometric/measurement; tool inventory, not intervention effects)
Higham et al. (37)	Systematic review	Assessment tools/measurement	Simulated and real clinical environments	Tool identification; development processes; validity/usability; rater training reporting	Yes (psychometric/measurement)
Dewolf et al. (24)	Systematic review	Training effectiveness	ALS simulation	NTS training effects on NTS performance and simulated process metrics (completion time); retention 3–6 months	Yes (training/process)
Bennett et al. (33)	Scoping review	Role requirements	Paramedicine; mixed/unspecified	Catalog of desirable NTS for paramedics	Limited/No (mapping of competencies)
Evans et al. (25)	Scoping review using PRISMA-ScR and Arksey & O'Malley's framework	Framework/Conceptual (taxonomy)	*Ad hoc* prehospital, ED, trauma resuscitation (mixed)	Taxonomy/terminology for team-related NTS concepts; measurement limitations	Limited/No
Hu et al. (38)	Systematic review	Framework/Conceptual (competency frameworks)	Emergency/disaster environments (all-hazards)	Framework characteristics; domain/terminology variability	Limited/No (framework synthesis; not intervention effects)
Tan et al. (30)	Scoping review	Role requirements	Acute medical emergencies (training/practice context)	NTS required for junior doctors; deficits linked to errors/delays (narrative synthesis)	Limited/No
Gawronski et al. (31)	Systematic review	Assessment tools/measurement	High-fidelity simulation (HFS)	Domains/item structures; psychometric properties; recommendation for TEAM in HFS	Yes (psychometric/measurement)
Stevenson et al. (28)	Scoping review	Assessment tools/measurement (T-NOTECHS)	Trauma/resuscitation; observational/video-linked contexts; real-time clinical needs highlighted	Development process; sensibility/reliability/validity evidence; need for real-time clinical validation	Yes (psychometric/measurement)
Alexander et al. (22)	Systematic review	Assessment tools/measurement	Video-based assessment of ED resuscitation teams	Validity/usability of video review tools; tool recommendation (T-NOTECHS)	Yes (psychometric/measurement/ usability)
Cooper et al. (23)	Mapping review	Assessment tools/measurement and training data (TEAM applications)	Simulation and real-life emergency exposure (as reported)	TEAM use across settings; improvement in TEAM-rated NTS; benchmarking	Yes (training/process) (also measurement)
Zhang (36)	Narrative review	Training design/context (simulation literature)	Emergency medicine simulation	Categorization by NTS domains; research design observations; standardization issues	Limited/No (predominantly narrative synthesis)
Jaspers et al. (35)	Scoping review	Training design (escape rooms)	Acute care training contexts	Design characteristics; relationship to learning outcomes; reporting gaps	Limited/No (insufficient evidence for effectiveness by design features)
Kang et al. (34)	Systematic review	Assessment tools/measurement (ANTS)	Anesthesia training/practice contexts	Validity/reliability/application of ANTS; relevance for safety education	Yes (psychometric/measurement)
Rheinberger et al. (32)	Scoping review	Role requirements	ED resuscitation nursing	Training needs; role description/scope gaps; competence maintenance needs	Limited/No
Sorensen et al. (27)	Narrative review	Stress/team performance	Interprofessional healthcare teams (mixed)	Stress impacts on team performance; protective factors (communication/shared mental models)	Limited/No

**Primary focus** indicates the main thematic focus of each review. **Setting** refers to the main context of evidence synthesis as reported in the review (e.g., simulation, video-based assessment, or clinical practice). **Main outcomes synthesized** describe the type of outcomes summarized in the review (e.g., behavioral ratings, training outcomes, or role competencies). **Quantitative outcome data** indicate whether the review reported measurable quantitative evidence (e.g., psychometric properties or measurable training effects).

### 3.2 Methodological quality of included reviews

The methodological quality of the included reviews varied. Based on the AMSTAR 2 assessment, four systematic reviews were rated as having moderate confidence and two were rated as having low confidence. No review met the criteria for high confidence. Common methodological limitations among systematic reviews included the absence of a prospectively registered protocol, incomplete reporting of excluded studies, and limited or inconsistent assessment of risk of bias in the included primary studies. Scoping and narrative reviews demonstrated variable reporting quality, as assessed using the PRISMA-ScR checklist and the SANRA instrument, respectively. Overall, the evidence base exhibited heterogeneity in methodological rigor. This variability was considered during data synthesis and informed a cautious interpretation of the findings.

### 3.3 Synthesis of evidence

This section provides a structured synthesis of the findings from the included reviews. The distribution of review focus, study settings, outcome types, and quantitative data is summarized in Table 4. For clarity, findings are presented under seven themes: (1) conceptual foundations of NTS; (2) study designs and methodological approaches; (3) tools for NTS assessment; (4) training strategies and educational interventions; (5) role-related NTS requirements; (6) reported outcome measures and quantitative data; and (7) impact on team performance and patient-related outcomes.

#### 3.3.1 Conceptual foundations of NTS

Conceptual and competency-based approaches to NTS were synthesized in three reviews (25, 29, 38). Carne et al. (29) described crisis resource management (CRM) as a key conceptual framework in emergency medicine, emphasizing leadership, communication, situational awareness, task allocation, and error management as behaviors relevant to safe team performance. Evans et al. (25) reviewed *ad hoc* prehospital, emergency department, and trauma resuscitation teams, identified inconsistent use of team-related terminology across the literature, and proposed a taxonomy to clarify how NTS-related constructs are conceptualized in resuscitation settings. Hu et al. (38) synthesized competency frameworks for emergency and disaster health professionals and reported marked differences in the number, naming, and organization of competency domains across frameworks. The three reviews describe different approaches used to define NTS in healthcare and report differences in terminology, framework structure, and conceptual scope (25, 29, 38). They also report differences in domain terminology, framework structure, and conceptual scope, which limit direct comparison of NTS domains across studies (25, 38).

#### 3.3.2 Study designs and methodological approaches

Six systematic reviews synthesized evidence on NTS training, assessment tools, or competency frameworks (22, 24, 31, 34, 37, 38). Seven scoping or mapping reviews aimed to map the existing literature and identify research gaps related to NTS roles, terminology, and training approaches (23, 25, 28, 30, 32, 33, 35). Four narrative or critical reviews discussed conceptual models, stress, or broader theoretical perspectives on NTS in healthcare teams (26, 27, 29, 36). Table 4 summarizes the distribution of review designs, study settings, and outcome focus.

Observer-based assessment using behavioral rating instruments was a prominent methodological approach in the literature. Six reviews synthesized tools designed to evaluate teamwork, leadership, communication, and decision-making in clinical or simulated environments (22, 23, 26, 28, 31, 37). For example, Higham et al. (37) synthesized observer-based assessment tools used in simulated and clinical settings, while Alexander et al. (22) examined video-based evaluation of NTS in emergency department resuscitation teams. Jepsen et al. (26) reviewed instruments designed to assess both individual- and team-level NTS in healthcare.

Simulation-based training interventions were reported in 4 of the 17 included reviews (24%) (23, 24, 35, 36). These studies primarily evaluated simulation-based educational strategies aimed at improving team behaviors and non-technical performance. Dewolf et al. (24) examined NTS-focused training interventions incorporated into advanced life support simulations. Jaspers et al. (35) analyzed escape-room–based crisis resource management and teamwork training in acute care education. Cooper et al. (23) mapped the application of the Team Emergency Assessment Measure (TEAM) in emergency team training and reported improvements in TEAM-rated NTS following repeated simulation or emergency exposure. Zhang (36) synthesized simulation studies in emergency medicine and categorized the NTS domains addressed in training. Reported outcomes mainly included observer-rated team behaviors, training performance indicators, and educational outcomes rather than patient-level clinical outcomes (23, 24, 35, 36). Qualitative approaches such as thematic or framework analysis were also used to identify key NTS domains and behavioral markers (30, 38).

#### 3.3.3 Tools for non-technical skills assessment

Observer-based instruments were the primary approach used to assess NTS across the included reviews. Application settings for NTS assessment tools were most frequently reported in simulation or high-fidelity simulation contexts (6/17 reviews, 35%) (23, 24, 31, 35–37), followed by video-based assessment (2/17, 12%) (22, 28). Explicit reporting of implementation in routine real-time clinical environments was less common (2/17, 12%) (23, 37).

Among the identified instruments, the Team Emergency Assessment Measure (TEAM) was the most frequently reported tool across the included reviews and was applied to diverse clinical teams, including adult, pediatric, obstetric, and student groups (23). Evidence synthesized in systematic reviews indicated acceptable psychometric properties for TEAM, particularly in simulation-based settings (31). The Trauma Non-Technical Skills scale (T-NOTECHS) was commonly used in trauma and resuscitation scenarios and frequently applied in video-based evaluation of team performance (22). Its behavioral structure aligns with time-critical and task-focused team activities, although supporting evidence primarily derives from observational or simulated environments rather than routine clinical practice. Specialty-specific instruments such as Anesthetists' Non-Technical Skills (ANTS), Observational Teamwork Assessment for Surgery (OTAS), and Non-Technical Skills for Surgeons (NOTSS) were originally developed for anesthesia and surgical settings. Reviews reported that these instruments provide structured behavioral markers that may be transferable to broader acute care contexts, although adaptation requires attention to contextual relevance and rater training (28, 34, 37).

Differences in instrument development, reporting of validity evidence, and descriptions of rater training were noted in reviews of NTS assessment instruments (26, 28, 31, 34, 37). For example, Higham et al. (37) and Jepsen et al. (26) highlighted variability in the development and validation processes of behavioral rating tools. Alexander et al. (22) and Stevenson et al. (28) also noted limitations in the available evidence supporting specific instruments used for trauma or resuscitation team assessment.

#### 3.3.4 Training strategies and educational interventions

Four reviews synthesized evidence on educational interventions aimed at improving NTS in acute care teams (23, 24, 35, 36). Training strategies most frequently involved team-based simulation combined with structured debriefing and repeated scenario exposure (24, 36). Simulation-based training programmes were designed to allow teams to practice communication, leadership, task coordination, and shared situational awareness in time-critical scenarios (24, 36).

Evidence from simulation-based advanced life support training indicated that NTS-focused instruction improved observable team behaviors during simulated cardiac arrest management and reduced scenario completion time, suggesting improved coordination and decision-making under pressure (24). Similarly, synthesis of studies using the Team Emergency Assessment Measure (TEAM) showed that repeated exposure to simulation or real-life emergency events was associated with higher TEAM scores, reflecting improvements in leadership, communication, and task management within emergency teams (23). Simulation-based education in emergency medicine also consistently targeted core NTS domains, particularly teamwork, communication, leadership, and decision-making, which were addressed through scenario-based practice and reflective debriefing (36). Alternative formats such as escape room–based crisis resource management training were explored in the literature, but inconsistent reporting of design features and learning objectives limited conclusions about which components contributed to learning effectiveness (35).

#### 3.3.5 Role-related non-technical skill requirements

Role-specific NTS requirements were synthesized in three reviews (3/17, 18%) focusing on junior doctors (30), paramedics (33), and emergency department resuscitation nurses (32). Teamwork and communication were identified as core competencies across these professional groups. Tan et al. (30) reported that junior doctors require behaviors such as timely escalation, help-seeking, task prioritization, and speaking up across hierarchical structures during acute care situations. Bennett et al. (33) highlighted coordination, communication, and situational awareness as key NTS for paramedic practice. Rheinberger et al. (32) described anticipatory task management, coordination with the resuscitation team, and proactive team support behaviors as essential competencies for emergency department resuscitation nurses.

#### 3.3.6 Reported outcome measures and quantitative data

Quantitative reporting of training effects on NTS was limited across the 17 included reviews. Two reviews (23, 24) reported extractable quantitative outcomes, while most others provided descriptive summaries without standardized effect estimates. Where quantitative data were reported, NTS-focused training, most often simulation-based training with structured debriefing, was associated with improvements in observer-rated team performance and process-related indicators.

Cooper et al. (23) synthesized 22 primary studies that applied the Team Emergency Assessment Measure (TEAM) and reported measurable improvements in team performance following structured team training. In some cases, TEAM global scores increased from approximately 6.0 to 9.0 after training. Effect sizes reported in individual studies ranged from moderate to large, with examples including Cohen's d values of 0.61 to 1.21. The review also described differences between experienced clinical teams and student teams, with TEAM performance ratings reported at approximately 90% for experienced teams compared with about 38% for student teams. Dewolf et al. (24) reported quantitative outcomes from advanced life support simulations that incorporated NTS-focused training. These studies described reductions in the time required to complete simulated cardiac arrest scenarios and shorter times to key clinical actions after training. Some primary studies included follow-up data indicating that improvements in team behavior were maintained for approximately 3 to 6 months after training.

No included review reported pooled meta-analytic effect estimates for training effectiveness. The quantitative evidence available in this umbrella review therefore reflects measurable outcomes reported in primary studies and summarized at review level rather than pooled effect estimates (Supplementary Additional File 2).

#### 3.3.7 Impact of NTS on team performance and patient-related outcomes

Reviews of NTS in acute care settings reported improvements in team performance indicators such as coordination, communication quality, adherence to protocols, and task completion time, particularly in resuscitation and other time-sensitive scenarios (23, 24, 27). These outcomes were commonly used as indicators related to patient safety goals (23, 24). Direct measures of patient safety, such as observed clinical error rates, preventable harm, or patient outcomes in real-world emergency department settings, were rarely reported. For example, Dewolf et al. (24) reported that structured NTS components incorporated into advanced life support simulations improved team behaviors and reduced time to key clinical actions, with some studies reporting persistence of behavioral improvements for several months. Sorensen et al. (27) synthesized evidence showing that stress negatively affected team processes and identified communication and shared mental models as factors associated with more effective team performance. Kang et al. (34) reviewed the Anesthetists' Non-Technical Skills (ANTS) framework as an instrument used to assess safety-related competencies and noted that further clinical validation in routine practice settings is still required.

## 4 Discussion

### 4.1 Simulation-based education as the dominant training context

Across the included reviews, simulation-based education emerged as the dominant context for both NTS training and assessment. Most training-related evidence synthesized in this umbrella review originated from simulation-based or educational settings rather than routine clinical environments. High-fidelity team simulations and structured debriefing were frequently used to examine communication, teamwork, leadership, and decision-making under conditions intended to approximate clinical complexity and time pressure. This pattern aligns with the distribution of settings reported across the included reviews (Table 4) and was particularly evident in reviews focusing on team-based simulation and structured debriefing approaches (23, 24, 37).

### 4.2 Evidence for team-level performance improvements

Evidence synthesized in included reviews indicates that NTS training is associated with improvements in team behaviors and process-related performance indicators in acute care settings, particularly in resuscitation and other time-critical scenarios (24, 31, 34, 37). Improvements were most commonly observed in observer-rated teamwork, communication, leadership behaviors, and completion time for simulated emergency tasks. Emergency departments rely heavily on interprofessional collaboration, and reviews of teamwork and crisis resource management frameworks emphasized the role of NTS in facilitating coordination, information sharing, and shared decision-making among multidisciplinary teams (26, 29).

Although improvements in behavioral ratings and time-based performance indicators were reported, the strength of quantitative evidence remains limited (24, 39), and none conducted pooled meta-analyses of training effectiveness. Consequently, the available quantitative evidence reflects review-level reporting rather than pooled effect estimates.

### 4.3 Assessment instruments and clinical applicability

A further contribution of this review lies in mapping the range of assessment instruments used to evaluate NTS across acute care contexts. Tools such as TEAM and T-NOTECHS were frequently reported, particularly in simulation-based and video-assisted assessments (22, 23, 31). Several specialty-specific instruments, including ANTS and NOTSS, were developed within anesthesia and surgical domains and may offer transferable behavioral markers for broader acute care environments.

However, many instruments were validated primarily in controlled or specialty-specific contexts. Variability in tool selection, reporting of validity evidence, and description of rater training complicates comparison across studies and settings. These factors highlight the need for careful contextual justification when selecting NTS assessment tools for emergency department use.

### 4.4 Transferability to emergency department practice

To interpret the relevance of the findings for emergency department practice, transferability was considered across three analytical dimensions: contextual alignment, measurement feasibility, and outcome relevance. Contextual alignment referred to whether the original evidence reflected the time pressure, frequent interruptions, parallel task demands, and rapidly changing team composition typical of emergency care. Measurement feasibility concerned whether assessment tools could be reliably applied in the intended setting, such as real-time clinical observation, video review, or simulation, and whether rater training and calibration procedures were adequately described. Outcome relevance referred to whether reported outcomes extended beyond behavioral ratings and process indicators to include clinical errors, preventable harm, or other clinically meaningful outcomes in routine practice. Where evidence was predominantly simulation-based or observational, implications for emergency department practice were interpreted cautiously, and tool use was regarded as informative for team performance evaluation rather than as direct evidence of clinical effectiveness.

### 4.5 Implementation challenges and barriers

Implementation challenges were also evident across the reviewed literature. Reported barriers included resource constraints, staffing pressures, hierarchical team structures (27), and limited opportunities for reinforcement in everyday clinical work (31). These factors are particularly salient in emergency departments, where operational demands may restrict the feasibility of repeated training and formal assessment (22). Effective implementation therefore requires not only validated tools and training strategies, but also organizational support, leadership engagement, and integration within existing quality and safety frameworks.

### 4.6 Global context and equity considerations

The evidence base synthesized in this umbrella review is predominantly derived from high-income countries, with limited representation from low- and middle-income settings. This imbalance restricts the generalisability of findings and highlights the need for frugal innovation in NTS education. While high-fidelity simulation is widely considered an effective educational approach in resource-rich settings, future research should evaluate low-cost, high-impact training modalities, such as peer-led coaching or low-resource tabletop exercises, that can be sustainably implemented in LMICs without reliance on expensive technology. Such context-sensitive approaches are essential to ensure that NTS frameworks are not only culturally adaptable but also economically viable for global emergency medicine.

### 4.7 Overall interpretation

Overall, this umbrella review reinforces the importance of NTS in supporting team processes in acute care environments. Behavioral and process-level improvements are consistently reported, particularly within structured training environments. However, translating these improvements into measurable patient-centered clinical outcomes remains challenging, reflecting both methodological constraints and the complexity of evaluating behavioral interventions within dynamic emergency care systems. Strengthening methodological rigor, harmonizing outcome reporting, and prioritizing real-world implementation research will be essential to advance the field.

## 5 Strengths and limitations

Several limitations of this review should be acknowledged. The inclusion of English-language publications only may have resulted in the omission of relevant evidence and introduced language bias. In addition, although 17 reviews were synthesized, most did not report pooled meta-analytic estimates or standardized effect sizes, which limits the ability to quantify effect sizes or draw definitive conclusions regarding the effectiveness of NTS interventions. The underlying evidence base was also dominated by observational and simulation-based studies, which may not fully capture the complexity, constraints, and competing priorities of routine clinical practice in emergency departments. Furthermore, most included reviews originated from high-income countries, restricting the applicability, and transferability of findings to low- and middle-income healthcare settings.

Notwithstanding these limitations, this umbrella review offers a structured synthesis and critical appraisal of review-level evidence on the assessment, training, and implementation of NTS across acute care contexts. By explicitly examining the relevance and limitations of the available evidence for emergency department practice, the review clarifies which aspects are currently supported by existing research and highlights important gaps that warrant further investigation, particularly with respect to real-world clinical outcomes and contextual transferability.

## 6 Implications for emergency clinical practice

The available evidence indicates that structured NTS education is associated with improvements in team behaviors and process-related performance indicators, particularly within simulation-based and educational contexts relevant to emergency care. On this basis, healthcare institutions may consider incorporating NTS principles into undergraduate, postgraduate, and continuing professional development programmes, considering local resources, workforce needs, and organizational priorities.

Because much of the existing evidence is derived from simulation-based environments, implementation in routine clinical settings should be accompanied by structured evaluation and monitoring. Institutions adopting NTS training programmes could integrate behavioral assessment tools, structured debriefing practices, and ongoing team performance feedback into existing quality improvement and patient safety systems. Evidence from low- and middle-income countries remains limited. Further context-specific adaptation and empirical evaluation will be required before broader implementation can be supported.

## 7 Recommendations for future research

Several important gaps in the current literature warrant further investigation. Although many studies examine the effects of NTS training on individual and team performance, few assess long-term skill retention or transfer to routine clinical practice. Longitudinal and multicentre studies are needed to evaluate sustainability and contextual influences on implementation (23).

Future research may also explore the use of digital technologies, including artificial intelligence and machine learning, to support performance feedback, behavioral assessment, and training evaluation. In addition, greater emphasis on studies conducted in low- and middle-income countries is needed to understand how NTS frameworks and training approaches can be adapted to different healthcare systems and resource environments.

## 8 Conclusion

This umbrella review highlights the importance of NTS for effective teamwork in emergency care. Evidence synthesized across the included reviews indicates that NTS-focused education is consistently associated with improvements in observed team behaviors and process-related performance indicators, particularly within simulation-based training environments. Among the available assessment tools, the TEAM instrument is frequently applied for structured evaluation and feedback during simulation-based team training, while T-NOTECHS has relatively stronger support in trauma and resuscitation-focused observational contexts.

However, a translational gap remains between training environments and routine clinical practice, and direct evidence linking NTS interventions to improvements in patient outcomes in emergency department settings remains limited. Future research should prioritize real-world implementation studies, methodological standardization, and evaluation of the sustainability of NTS training across diverse healthcare systems.

## Data Availability

The original contributions presented in the study are included in the article/Supplementary material, further inquiries can be directed to the corresponding author.
