# Supplementary material for: Non-technical skills in acute care: an umbrella review of assessment, training, and implications for emergency department practice
Source: Front Med (Lausanne). 2026 Apr 28;13:1791714. doi: 10.3389/fmed.2026.1791714 (PMC13160740; doi:10.3389/fmed.2026.1791714)
Supplement: Supplementary file 1 [file Table_1.docx]

## **Additional file 1. Selection of databases and search strategy**

To capture literature relevant to non-technical skills and emergency care, we searched five major electronic databases from inception to February 2025: PubMed (MEDLINE), Scopus, Cochrane Library, Web of Science, and EBSCOhost (CINAHL, APA PsycInfo, and Academic Search Complete). Together, these databases cover medicine, nursing, psychology, and allied health disciplines. Citation tracking and reference list screening were also used to supplement the database searches.

The initial search strategy was developed by YKZ and refined through team discussion. A professional librarian with expertise in systematic review searching reviewed and optimised the search terms, Boolean operators, and database-specific adaptations to enhance sensitivity and methodological rigour.

**Supplementary material--Search equations**

| **Database** | **Search equations** | **Filters** |
| --- | --- | --- |
| PubMed | ((((((non-technical skills) OR (human factors)) OR (soft skill)) OR (Crisis Resource Management)) OR (crew resource management)) AND ((((((Emergency Medicine) OR (emergency department)) OR (acute care)) OR (Critical Care)) OR (trauma)) OR (resuscitation))) AND ((((((teamwork) OR (communication)) OR (leadership)) OR (situational awareness)) OR (task management)) OR (decision making)) | Reviews |
| Scopus | ( ( TITLE-ABS-KEY ( non technical skills ) OR TITLE-ABS-KEY ( soft skill ) OR TITLE-ABS-KEY ( Crisis Resource Management ) OR TITLE-ABS-KEY ( crew resource management ) ) ) AND ( ( TITLE-ABS-KEY ( emergency department ) OR TITLE-ABS-KEY ( acute care ) OR TITLE-ABS-KEY ( Critical Care ) OR TITLE-ABS-KEY ( intensive care units ) OR TITLE-ABS-KEY ( trauma ) OR TITLE-ABS-KEY ( resuscitation ) ) ) AND ( ( TITLE-ABS-KEY ( teamwork ) OR TITLE-ABS-KEY ( communication ) OR TITLE-ABS-KEY ( leadership ) OR TITLE-ABS-KEY ( situational awareness ) OR TITLE-ABS-KEY ( task management ) OR TITLE-ABS-KEY ( decision making ) ) ) | Reviews |
| Web of Science | Non-Technical Skills OR Soft skills OR Crew Resource Management OR Crisis Resource Management OR human factors (Topic) AND Teamwork OR Communication OR Leadership OR Situational awareness OR Task management OR Decision making (All Fields) AND Emergency Medicine OR Emergency department OR Acute care OR Critical Care OR Trauma OR Resuscitation (All Fields) and Review Article (Document Types) | Reviews |
| Cochrane Library | (non-technical skills):ti,ab,kw OR (human factors):ti,ab,kw OR (soft skill):ti,ab,kw OR (Crisis Resource Management):ti,ab,kw OR (crew resource management):ti,ab,kw AND (teamwork):ti,ab,kw OR (communication):ti,ab,kw OR (leadership):ti,ab,kw OR (situational awareness):ti,ab,kw OR (task management):ti,ab,kw AND ("emergency medicine"):ti,ab,kw OR (emergency department):ti,ab,kw OR (acute care):ti,ab,kw OR ("critical care"):ti,ab,kw OR (resuscitation):ti,ab,kw | - |
| EBSCOhost | (Non-Technical Skills OR Soft skills OR Crew Resource Management OR Crisis Resource Management OR human factors) AND (Teamwork OR Communication OR Leadership OR Situational awareness OR Task management OR Decision making) AND (Emergency Medicine OR Emergency department OR Acute care OR Critical Care OR Trauma OR Resuscitation) | Human and English |
