# Supplementary material for: Non-technical skills in acute care: an umbrella review of assessment, training, and implications for emergency department practice
Source: Front Med (Lausanne). 2026 Apr 28;13:1791714. doi: 10.3389/fmed.2026.1791714 (PMC13160740; doi:10.3389/fmed.2026.1791714)
Supplement: Supplementary file 2 [file Table_2.docx]

**Additional file 2**

**Supplementary material--Examples of quantitative training effects reported in included reviews**

| **Review (year)** | **Setting / Participants** | **Training type** | **Outcome metric** | **Reported quantitative effect** | **Follow-up** |
| --- | --- | --- | --- | --- | --- |
| Dewolf et al. (2020) | Advanced Life Support simulation; students to experts | Simulation based ALS with explicit NTS components and debriefing | Time based process indicators; team behaviour ratings | Reported “reduction in time required to complete a simulated cardiac arrest” and shorter times for critical initial steps; some studies reported persistence of behavioural effects for 3 to 6 months | Up to 3 to 6 months (reported in some studies) |
| Cooper et al. (2023) | Emergency teams (resuscitation, MET, students vs clinicians) | Simulation based training and repeated practice; some live emergencies | TEAM global/team domain scores; proportion style ratings; effect sizes in primary studies | Reported TEAM performance ratings ranging from ~90% (experienced clinical teams) to ~38% (students); examples of pre post improvement such as mean global score from 6.0 to 9.0 (reported as ~90%); review also reports moderate intervention effects and provides examples of large training effects from primary studies (Cohen’s d values reported in included studies such as d=0.61, d=1.10, d=1.21) | Some studies included follow up, including 6 months in at least one example study |
